# Elucidation of the reaction mechanism for the synthesis of ZnGeN_2_ through Zn_2_GeO_4_ ammonolysis†

**DOI:** 10.1039/d1sc00328c

**Published:** 2021-05-13

**Authors:** Zhenyu Wang, Daniel Fritsch, Stefan Berendts, Martin Lerch, Joachim Breternitz, Susan Schorr

**Affiliations:** Helmholtz-Zentrum Berlin für Materialien und Energie GmbH, Department Structure and Dynamics of Energy Materials Hahn-Meitner-Platz 1 14109 Berlin Germany joachim.breternitz@helmholtz-berlin.de susan.schorr@helmholtz-berlin.de; Freie Universität Berlin, Department Geosciences Malteserstraße 74-100 12249 Berlin Germany; Technische Universität Berlin, Fakultät II, Institut für Chemie Straße des 17. Juni 135 10623 Berlin Germany; Universität Potsdam, Mathematisch-Naturwissenschaftliche Fakultät, Institut für Chemie Karl-Liebknecht-Straße 24-25 14476 Potsdam Germany

## Abstract

Ternary II–IV–N_2_ materials have been considered as a promising class of materials that combine photovoltaic performance with earth-abundance and low toxicity. When switching from binary III–V materials to ternary II–IV–N_2_ materials, further structural complexity is added to the system that may influence its optoelectronic properties. Herein, we present a systematic study of the reaction of Zn_2_GeO_4_ with NH_3_ that produces zinc germanium oxide nitrides, and ultimately approach stoichiometric ZnGeN_2_, using a combination of chemical analyses, X-ray powder diffraction and DFT calculations. Elucidating the reaction mechanism as being dominated by Zn and O extrusion at the later reaction stages, we give an insight into studying structure–property relationships in this emerging class of materials.

## Abstract 2

Combining chemical analyses with detailed structural work, we decipher the reaction pathway in the ammonolysis of Zn_2_GeO_4_ towards ZnGeN_2_.

## Graphical abstract

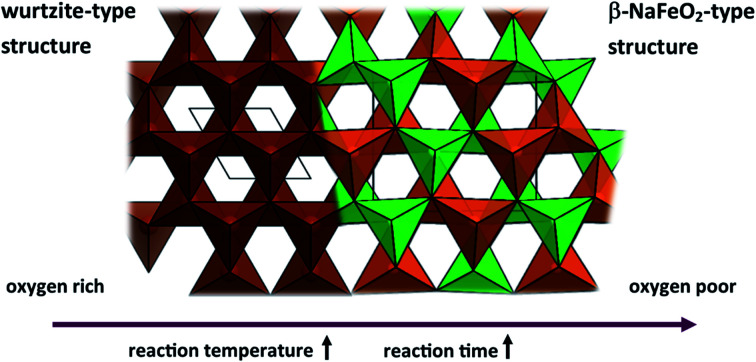

## Introduction

Most of the promising candidates as alternative solar cell materials struggle from severe problems concerning elemental abundance and toxicity. This prominently includes halide perovskites that predominantly contain toxic lead,^1–3^ but also III–V materials due to the low abundance of In and Ga.^4,5^ In response to the increasing awareness of these “secondary” judgement parameters – as opposed to a pure focus on solar cell efficiencies – the problematic cations are being substituted by abundant elements. The cations in III–V nitride materials are trivalent, but there is a limited number of trivalent cations they could be replaced with. Instead, they can be replaced by a combination of divalent and tetravalent cations,^6^ a similar rationale that also stands behind the development of lead-free double perovskites.^7,8^

**Fig. 1:**
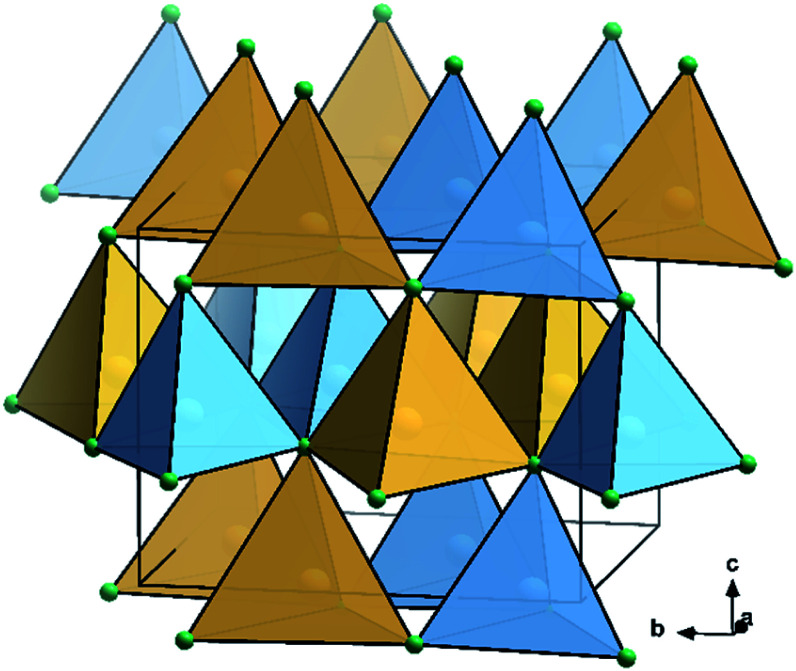
Crystal structure representation of ZnGeN_2_ in the β-NaFeO_2_-type.^15^ N: green, Ge: sky blue, Zn: canary yellow; coordination tetrahedra are drawn around the cations in the colours of the central atoms.

Particularly II–IV–N_2_ nitride materials have moved into the focus of research as they seemingly fulfil all the criteria as outlined above. While the binary nitrides AlN, GaN and InN all crystallise in the hexagonal wurtzite-type structure (space group *P*6_3_*mc*),^9,10^ the situation for the ternary compounds ZnGeN_2_ and ZnSnN_2_ is more complex. While ZnGeN_2_ is consistently reported to crystallise in the orthorhombic β-NaFeO_2_-type structure (space group *Pna*2_1_, Fig. 1), in which the Zn^2+^ and Ge^4+^ cations are ordered on different crystallographic sites, a variable degree of cation disorder was observed,^11^ up to the point where full disorder and a crystal structure in the wurtzite-type has been observed. As to what concerns ZnSnN_2_, no compelling experimental evidence for cation ordering has been observed so far,^12,13^ although numerous computational studies unanimously identified the β-NaFeO_2_-type structure as the thermodynamically stable crystal structure, similar to ZnGeN_2_.^14^

The situation becomes even more complex when taking oxygen into account: zinc germanium oxide nitrides (ZGON) exhibit a disordered wurtzite-type structure over a wide range of chemical compositions.^5,16–19^ This is insofar important as traces of oxygen are present in virtually any nitride material and it is thus important to disentangle the effect of oxygen on the cation disorder from exclusive cation disorder very carefully. For this, we studied the reaction of Zn_2_GeO_4_ as ternary oxide precursor with NH_3_ in order to obtain powder samples with a variable oxygen content. Using a model originally proposed by Bacher *et al.*,^19^ we studied the behaviour in the oxygen richer regime in a previous study, where a distinct separation of two competing processes was observed: (1) nitrogen inclusion on the one hand and (2) Zn loss on the other hand. In accordance to Bacher *et al.*, this can be formulated in two reaction steps:

1 Zn_2_GeO_4_ + 2*y*NH_3_ → Zn_2_GeO_4−3*y*_N_2*y*_ + 3*y*H_2_O

2 Zn_2_GeO_4_ + 2*x*/3NH_3_ → Zn_2−*x*_GeO_4−*x*_ + *x*/3Zn_3_N_2_ + *x*H_2_O

Zn_2_GeO_4_ + (2*x*/3 + 2*y*)NH_3_ → Zn_2−*x*_GeO_4−*x*−3*y*_N_2*y*_ + *x*/3Zn_3_N_2_ + (*x* + 3*y*)H_2_O

Herein, we explore the later stages of the overall reaction where the compound is approaching the stoichiometric nitride ZnGeN_2_. Through a combination of X-ray diffraction and chemical analyses, we are able to clarify the reaction pathway, which aids to understand the structural and electronic features of this class of compounds.

## Experimental section

### Syntheses

The zinc germanium oxide nitrides were synthesised through a two-step process as outlined earlier.^5,20^ Briefly, Zn_2_GeO_4_ was synthesised from binary ZnO (Fisher Scientific, 99%) and GeO_2_ (ACROS, 99.999%) at high temperatures and was then used as precursor in an ammonolysis reaction. For this, Zn_2_GeO_4_ was reacted in a silica glass reaction tube with 4.5 cm diameter. The samples were treated under a N_2_-flux of 1.5 l min^−1^ during heating (250 K h^−1^), and were reacted under a NH_3_ flux of 0.15 l min^−1^ (99.8%, AirLiquide) during the dwelling period. Time and temperature of the dwelling were varied between 10 h–20 h and 835 °C–910 °C, respectively, to produce powder samples of varying oxygen content. The exact reaction conditions of all samples are given in the ESI.†

### Characterisation

X-ray diffraction (XRD) data in a 2*θ* range from 15° to 140°were collected using a Bruker D8 Advance powder diffractometer with Cu-K_α1_ (*λ* = 1.54056 Å) and Cu-K _α2_ (*λ* = 1.54439 Å) radiation. The diffraction patterns were analysed using the Rietveld refinement method applying the FullProf Suite 3.0 software.^21^ The starting model for zinc germanium nitride in the orthorhombic β-NaFeO_2_-type structure was taken from Zhang *et al.*^22^ In the refinement, a linear interpolation background was used along with a Thompson–Cox–Hastings pseudo-Voigt profile with anisotropic line broadening correction through spherical harmonics in the Laue class *mmm*.^23^

X-ray fluorescence spectra (XRF) were collected using a Bruker M4 Tornado system with Rh-microfocus tube for the determination of the cation ratios. The tube voltage was set to 50 kV. Samples were pressed to pellets with 5 mm in diameter to avoid contamination when measuring in vacuum. Further, the pellets offer a flat surface for focusing in order to eliminate an undesired background. For each pellet, data on 6 different measuring points, at least, were collected with a collection time of 60 s per point.

Hot-gas extraction method was performed using a LECO TC-300/EF-300 instrument to determine O and N contents. Samples of approximately 10 mg were used for each independent measurement. The average value of three repeated measurements was taken as the final data with a relative error of 2%.

UV-VIS measurements were performed using a PerkinElmer LAMBDA 750S with a 100 mm integrating sphere in the range of 1000–250 nm and a step-width of 2 nm. Samples were measured in diffuse reflectance with the powders contained in silica glass cuvettes. The light absorption was estimated from reflection using the Kubelka–Munk function *F*(*R*) = (1 − *R*)^2^/2*R* (where *R* is the reflectance of the sample).^24^ The optical bandgap was then extracted using a Tauc-plot with [*F*(*R*) × *hν*]^2^ for a direct, allowed band gap.^25^

Density Functional Theory (DFT) calculations were performed utilising the Vienna *ab initio* simulations package (VASP 5.4.4)^26,27^ together with the projector-augmented wave (PAW) method.^28^ The 2 × 2 × 2 supercells of the primitive orthorhombic unit cell of ZGONs were constructed for the calculations. The supercells contain 128 atoms: 36 Zn, 28 Ge, 60 N and 4 O, according to the Zn/Ge ratio of 1.28 to reflect an experimentally accessed oxide nitride composition. The initial structural parameters, including lattice parameters and atomic positions were taken from the Rietveld refinement results of the X-ray diffraction pattern. Given the crystal structure of this experimentally accessed oxide nitride that is necessarily disordered, a random distribution of cations was generated using random shuffle^29^ function in python 3.8, to reflect the disordered cations arrangement, whereas atomic positions remained. Different oxygen-containing supercell models were built by accommodating all oxygen atoms in either [OZn_4_], [OZn_3_Ge_1_], [OZn_2_Ge_2_] or [OZn_1_Ge_3_] tetrahedra. The supercell structures were relaxed using the PBEsol^30^ functional until the forces on all atoms were below 0.1 eV Å^−1^. The lattice parameters were fixed during the optimisation of the atomic positions (*a* = 11.03078 Å, *b* = 12.83132 Å, and *c* = 10.38778 Å) in a Γ-point optimisation. A 2 × 2 × 2 Γ-centred *k*-point mesh was used for the subsequent total energy calculation using PBEsol. Further, Γ-point HSE06 (ref. 31) calculations were performed for total energies of the relaxed structures in comparison to the PBEsol values. Other parameters included a 500 eV cut-off energy for the plane-wave expansion, and a cut-off for the total energy convergence of 10^−6^ eV.

## Results

### Chemical composition of the samples

It is not straightforward to determine the four elements of zinc germanium oxide nitrides at the same time with the methods that are easily accessible. While XRF is most reliable for zinc and germanium, the quantisation of the oxygen and nitrogen contents are limited by this method. Instead, oxygen and nitrogen can be quantified using a combustive hot gas extraction method, which is specific to these elements and therefore does not allow the determination of the Zn and Ge contents. Therefore, both methods had to be combined to determine the complete composition of the respective samples.

It proved useful to employ the cation and anion ratios in our analysis, as they are easily accessible from the experimental characterisation: the XRF measurements yielded the atomic ratios of Zn and Ge directly, whereas the weight fractions obtained for O and N [*w*(O) and *w*(N) resp.] were converted using the respective molar masses [*M*(O) and *M*(N)]:^5^
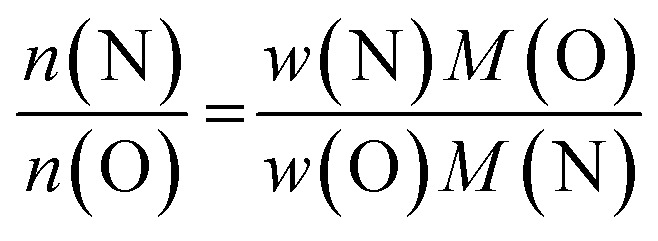


**Fig. 2:**
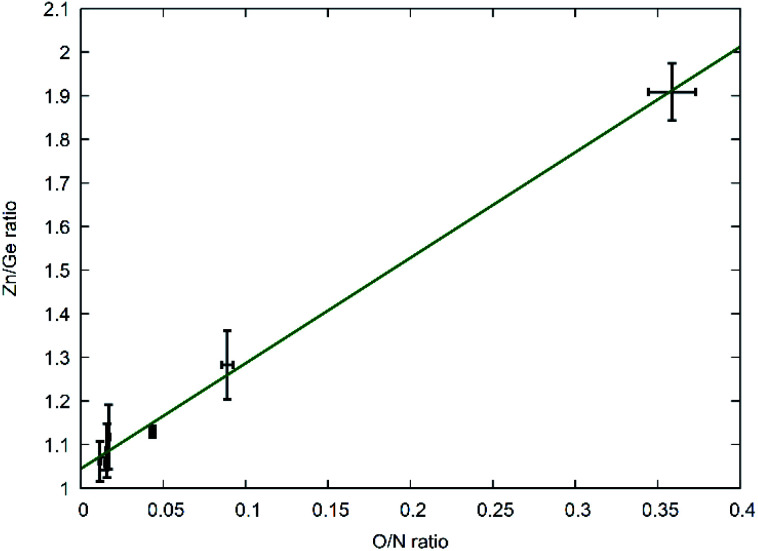
Experimentally determined Zn/Ge ratios against the experimentally determined O/N ratios. The green line shows a linear fit of the data points with *f*(*x*) = 2.42(7)*x* + 1.04(1).

This latter treatment is facilitating the analysis, as the exact composition of each individual compound is not known *a priori*. The ratio as defined above, however, does not depend on the knowledge of the exact composition and can hence be directly calculated on the basis of the experimentally determined values. A clear trend between the experimentally determined Zn/Ge ratio and the O/N ratio is evident throughout the samples (Fig. 2). Combining the Zn/Ge and O/N ratios, it is possible to calculate the overall composition using the general equation Zn_2*x*_GeO_4−*x*−3*y*_N_2*y*_ (Table 1).

**Table 1:** Experimentally determined Zn/Ge ratios and O/N ratios of the samples in this study together with the compositions as calculated from the formula Zn_2−*x*_GeO_4−*x*−3*y*_N_2*y*_. Full synthesis conditions may be found in the ESI

Sample number	Zn/Ge ratio	O/N ratio	Nominal composition
1	1.91(7)	0.36(1)	Zn_1.91_GeO_0.75_N_2.10_
2	1.28(8)	0.089(4)	Zn_1.28_GeO_0.18_N_2.07_
3	1.13(1)	0.043(1)	Zn_1.13_GeO_0.09_N_2.03_
4	1.12(7)	0.017(1)	Zn_1.12_GeO_0.03_N_2.05_
5	1.09(6)	0.016(1)	Zn_1.09_GeO_0.03_N_2.04_
6	1.06(1)	0.015(1)	Zn_1.06_GeO_0.03_N_2.02_
7	1.06(5)	0.011(1)	Zn_1.06_GeO_0.02_N_2.03_

The Zn/Ge ratio varies between 1 and 2, which is in line with the compositions of the boundary compounds: stoichiometric ZnGeN_2_ with Zn/Ge = 1 and Zn_2_GeO_4_ with Zn/Ge = 2. While the samples close to Zn/Ge = 1 also exhibit an O/N ratio near 0 – as expected for ZnGeN_2_ – the O/N ratio is only at ≈0.4 for Zn/Ge reaching to 2. This is in line with our prior findings that oxygen richer zinc germanium oxide nitrides preserve a Zn/Ge ratio close to 2, although they already contain notable amounts of nitrogen.

The sheer number of parameters that influence the composition of the compounds makes a straightforward analysis difficult. It is, however, evident that the higher the amount of starting materials is, the higher is the content of oxygen in the product. Also, the atmosphere under which the sample was cooled plays an important role in the resulting composition: when cooling under ammonia, the oxygen amount is considerably lower than cooling under nitrogen flow, which hints that the reaction continues at lower temperatures during the cooling period. Also, there is a number of samples with slightly different compositions, although they are made at nominally similar conditions, which is a clear sign for the complexity of the reaction and that the reaction conditions need to be controlled very carefully.

### Crystal structure of the zinc germanium oxide nitrides

All of the compositions showed single phase patterns with the exception of the oxygen poorest sample, where a miniscule inclusion of elemental Ge_3_N_4_ was found, which was also the reason not to extend the study further to longer reaction times and/or reaction temperatures. The crystal structure appears to be different for the oxygen-poor samples, which crystallise in the β-NaFeO_2_-type structure (space group *Pna*2_1_), and for the oxygen richer samples that appear to crystallise in the wurtzite-type structure (space group *P*6_3_*mc*). It should be noted that β-NaFeO_2_-type structure space group is a subgroup of the wurtzite-type structure and it is hence possible to perform the Rietveld refinements for all patterns in the lower symmetry β-NaFeO_2_-type crystal structure.^32^

**Fig. 3:**
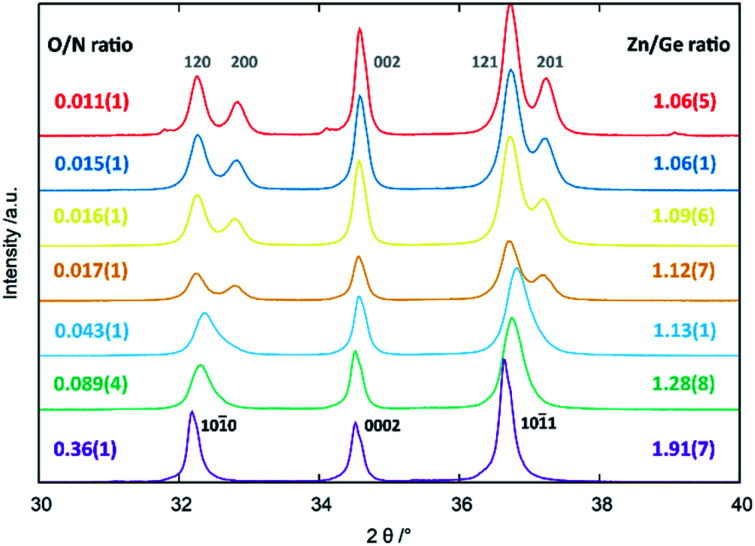
XRD patterns in the region of 30°–40° 2*θ* as a function of Zn/Ge and O/N ratios. The *hkl* indices according to the hexagonal wurtzite-type structure (black, bottom) and the *hkl* indices according to the orthorhombic β-NaFeO_2_-type structure (grey, top) are given in the figure.

**Fig. 4:**
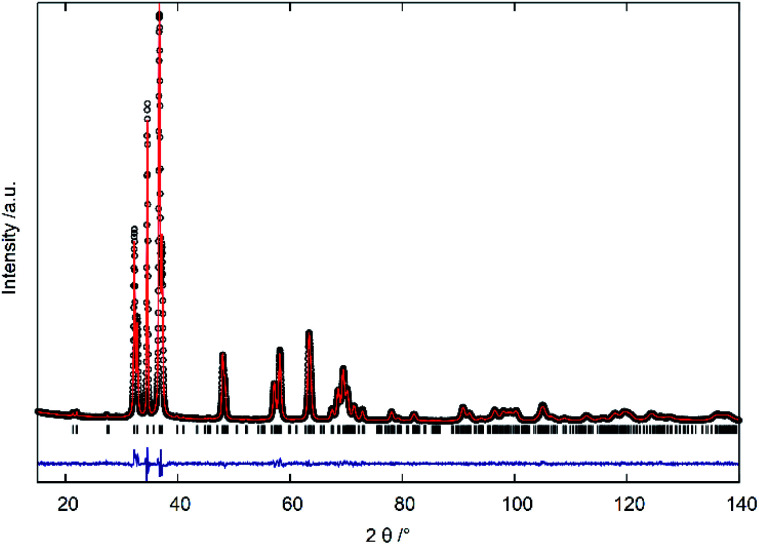
Plot of powder X-ray diffraction profile refinement with the Rietveld method: measured intensities (black circles), calculated profile (red line), difference between measured and calculated intensities (blue line) and calculated reflection positions (black ticks).

The three strongest groups of reflections between 30°–40° 2*θ* are most indicative for the transition from the hexagonal wurtzite-type structure to the orthorhombic β-NaFeO_2_-type structure (Fig. 3). The oxygen richer samples exhibit three reflections, in accordance to the hexagonal wurtzite-type structure. Still, the 101̄0 and 101̄1 reflections appear more and more asymmetric the lower the oxygen content becomes, until a very clear splitting appears, which is indicative of the β-NaFeO_2_-type structure. The 0002 reflection is, however, unaffected by the group–subgroup transition, as it does not split (002 reflection in the orthorhombic subgroup). Further, the Rietveld refinements (Fig. 4 and ESI†) were performed using an anisotropic line broadening correction as the 00*l* reflections appear systematically narrower than the remaining reflections, indicative of an anisotropic particle size.

**Fig. 5:**
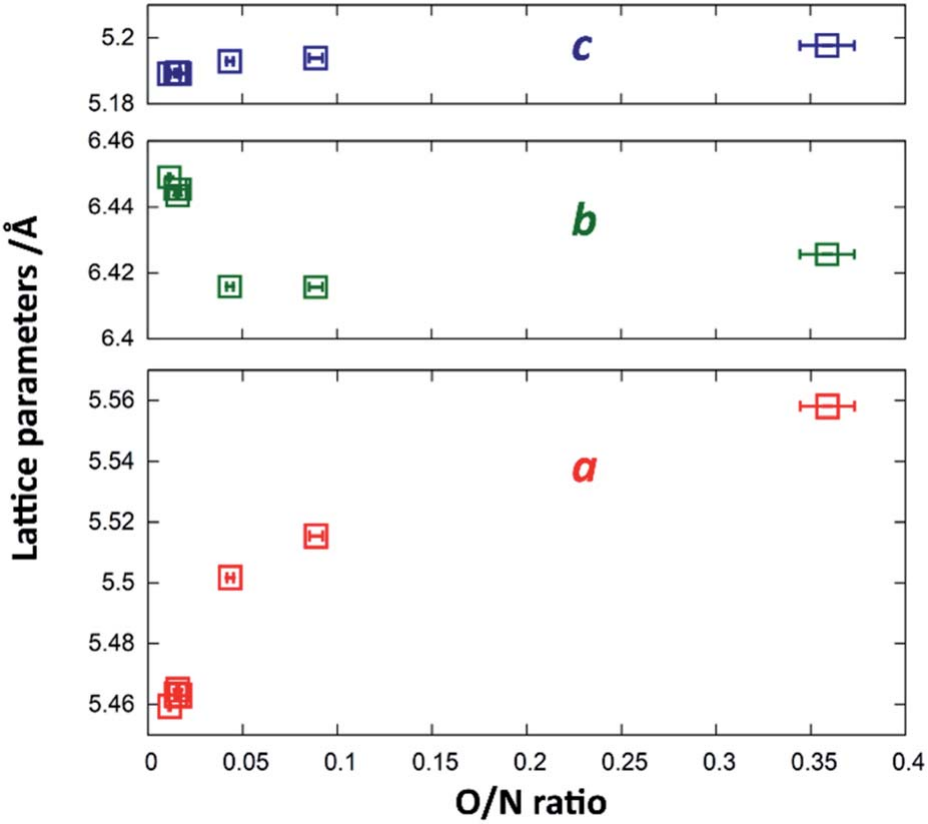
Change of the lattice parameters *a* (red), *b* (blue) and *c* (green) as a function of the O/N ratio.

Since Zn^2+^ and Ge^4+^ as well as O^2−^ and N^3−^ are formally isoelectronic, they are hardly distinguishable from each other using X-ray diffraction techniques. Therefore, the Rietveld refinements were mainly performed to confirm the overall crystal structure and to extract the lattice parameters. While the *c*-parameter remains largely unaffected over the entire composition range (Fig. 5), *a* and *b* vary significantly over the composition range. When approaching stoichiometric ZnGeN_2_, *i.e.* an O/N ratio of 0, the *a*-parameter shrinks more significantly than the *b*-parameter grows leading to an overall decrease in the unit cell volume. This can be rationalised by regarding the Shannon radii of the cations: *r*(Zn^2+^) = 0.6 Å and *r*(Ge^4+^) = 0.39 Å. The oxide nitrides contain a ratio of Zn/Ge that is above 1, but which reduces to 1 when approaching stoichiometric ZnGeN_2_. The share of larger Zn^2+^ cations, therefore, shrinks from oxygen richer oxide nitrides to ZnGeN_2_ and affects the volume in the same way.

In order to quantify the deviation of the observed crystal structure from an idealised wurtzite-type structure in a hexagonal unit cell, it is useful to compare the lattice parameters *a* and *b* as if they were in a hexagonal setting. According to the group–subgroup relationship between the hexagonal wurtzite-type structure and the orthorhombic β-NaFeO_2_-type structure,^32^ the orthorhombic distortion may be calculated as (*a*_h2_ − *a*_h1_)/*a*_h1_, where *a*_h*x*_ are the pseudo-hexagonal lattice parameters, which relate to the orthorhombic lattice parameters as *a*_h1_ = *a*_o_/√3 and *a*_h2_ = *b*_o_/2. This value is lies in a range between 0 for an ideal wurtzite-type structure and 2.27%, which is the value observed from DFT crystal structure optimisation.^33,34^

### UV-VIS measurements

**Fig. 6:**
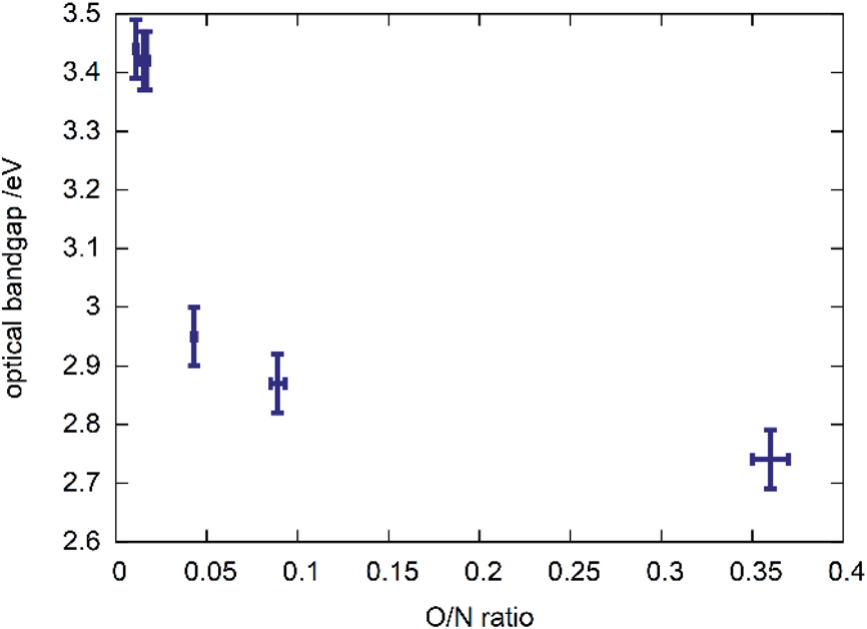
Optical bandgap as a function of the measured O/N ratio.

A clear trend can be observed between the chemical composition of the samples (represented by the O/N ratio in Fig. 6) and the bandgap in that the bandgap tends to decrease with rising O/N ratio. This relationship is, however, not linear and the samples with very low oxygen content show very similar optical bandgaps of ≈3.4 eV. Further investigations on the potential impact of cation order/disorder on this trend are currently ongoing. It is worth mentioning that a general parabolic relationship between bandgap and O/N ratio was observed in other recent study focussing on thin film syntheses of zinc germanium oxide nitrides, which show disorder of the cations.^35^ Finally, an excellent review by Schnepf *et al.* needs to mentioned, focussing on the influence of cation disorder on the band gaps in this class of materials.^36^

### DFT calculations

**Fig. 7:**
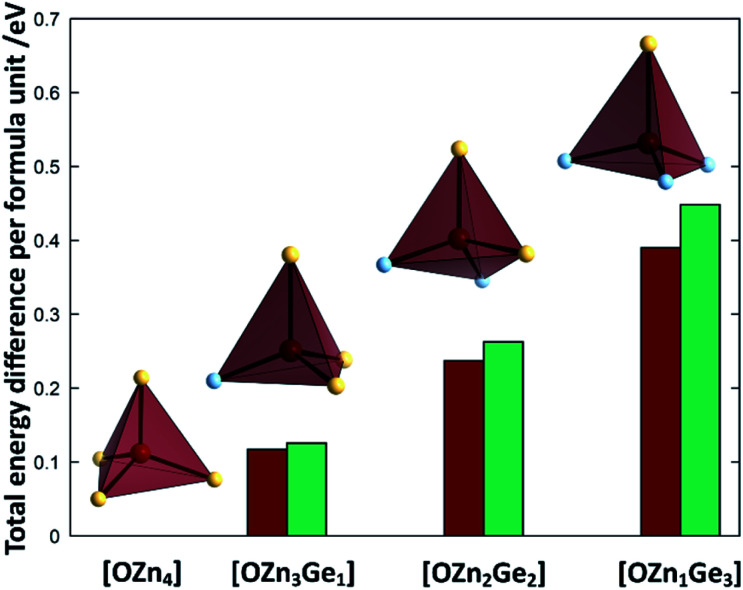
Differences of the total energies per formula unit of the different oxide nitride supercells as a function of the oxygen coordination environment. The values for 2 × 2 × 2 *k*-point grid PBEsol (red) and gamma-point HSE06 calculations (green) are given relative to the energy of the crystal structure, where oxygen is uniquely surrounded by Zn. The tetrahedra depict one representative tetrahedron for each coordination environment (O: bordeaux, Zn: canary yellow, Ge: sky blue).

To understand the reaction mechanism, we performed DFT calculations to probe, whether the arrangement of anions and cations are dependent of each other. For this, we compare the total energies of different arrangements within the same overall composition with each other (Fig. 7). The composition chosen reflects the composition at a Zn/Ge ratio of 1.28 and hence lies within the experimentally accessed parameter space.

It is very evident from the total energies of the different arrangements, that oxygen prefers to be surrounded by Zn rather than Ge. The total energies for the supercell containing uniquely Zn coordinated O is consistently the lowest throughout the functionals tested. The energy difference between the [OZn_4_] and the [OZn_3_Ge_1_] coordinations of 125.5 meV per f.u. (HSE06) would correspond to a thermal activation temperature of 1456 K, which is considerably above the reaction temperatures used in this study.

## Discussion

### Elucidation of the reaction mechanism

**Fig. 8:**
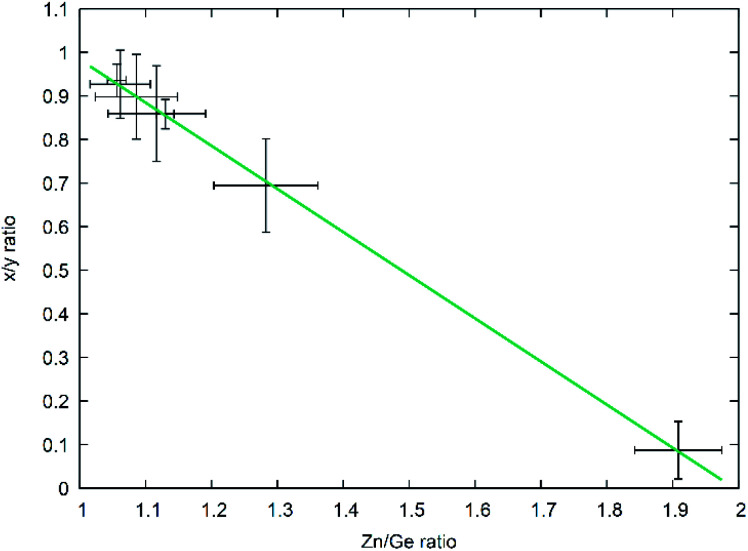
*x*/*y* ratio as a function of the Zn/Ge ratio throughout the composition range. The green line depicts a linear fit of the data.

As developed by Bacher,^19^ one can easily separate the overall reaction into the part-reactions (1) nitrogen inclusion and (2) Zn loss. As they both influence the composition of the product Zn_2−*x*_GeO_4−*x*−3*y*_N_2*y*_ in a different way – (1) acting upon *y* and (2) acting upon *x* – one can disseminate the contribution of each partial reaction from the chemical composition of the product. This does, in fact, allow to elucidate the reaction mechanism over the course of the reaction conditions studied and hence allow a more targeted synthesis approach. For this, it is useful to use the ratio of the parameters *x* and *y* as an indicator for the relative contribution of both part-reactions. It is evident that there is a very clear relationship between the *x*/*y* ratio and the Zn/Ge ratio (Fig. 8) with a general formula of the linear fit of *x*/*y* = 1.97(1) − 0.99(1)Zn/Ge. Since the relationship *x* = 2 − Zn/Ge is defined through the overall formula and taking the linear fit of the relationship between *x*/*y* and Zn/Ge into account, this essentially means that *y* ≈ 1, throughout the whole composition range in this study. This in turn means, that the nitrogen inclusion part reaction is essentially finished and only Zn loss proceeds through the reaction period. This essentially means that the simplified formula Zn_2−*x*_GeN_2_O_1−*x*_ can be used as a convenient first approximation to the accurate chemical composition, which can be determined on the basis of the Zn/Ge ratio.

It is important to put this finding in the perspective of the overall reaction: while we studied the later stages of the reaction herein, and for which these results are valid, the earlier stages of the reaction do not necessarily follow the same scheme. In a previous study,^5^ we produced zinc germanium oxide nitrides with higher oxygen contents that were crystallising in the wurtzite-type. In this oxygen richer regime, a clear transition in the reaction scheme could be observed, in that we obtained compositions containing variable amounts of nitrogen while still maintaining a Zn/Ge ratio close to 2 (and in fact sometimes even higher than 2 – a fact that is probably due to slight loss of Ge, potentially in the form of volatile GeO). For these earlier stages, the nitrogen inclusion reaction is not completed, and therefore implying a limitation of the model developed herein to the later stages of the reaction. Those are signified by a Zn/Ge ratio notably below 2. Given that the oxygen poorer compounds crystallising in the β-NaFeO_2_-type crystal structure are the relevant stage for potential cation order/disorder phenomena, we believe that the mechanistic description of this reaction period is most important for further understanding of the materials properties.

### Verification and implications of the reaction model

From a purely formal point of view, one can understand the simplified general formula Zn_2−*x*_GeN_2_O_1−*x*_ as a linear combination of the form *k*ZnGeN_2_ + (1 − *k*)ZnO, where *k* = 1/(2 − *x*). Purely formal in the sense that there is no evidence from the structural point of view that the compound is separated in ZnGeN_2_ and ZnO, but it forms a uniform body. This treatment is, nonetheless, useful as it allows determine the parameter *k* directly and independently over the cation (*k*_c_) and the anion ratios (*k*_a_) and thereby allows an effective way to probe the model for its validity. The parameters are defined with the following formulae, where *n*(*X*) are the respective molar amounts:*k*_c_ = *n*(Ge)/*n*(Zn)*k*_a_ = 1/(2 × [*n*(O)/*n*(N)] + 1)

**Fig. 9:**
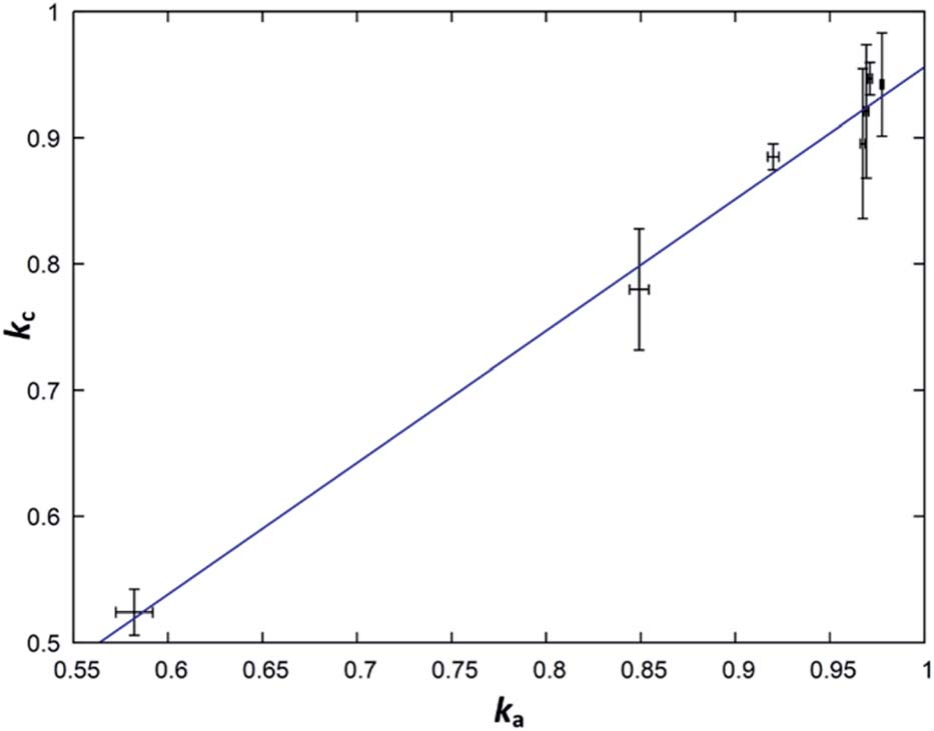
Plot of the parameter *k* as calculated from the cation ratio (*k*_c_) in dependence of the same parameter as calculated from the anions ratio (*k*_a_). The blue line signifies the linear fit *k*_c_ = 1.04(5)*k*_a_ − 0.08(5).

While the fit (Fig. 9) suffers from relatively large experimental errors, there is a clear trend between *k*_a_ and *k*_c_, which can be fit with a linear trend as *k*_c_ = 1.04(5)*k*_a_ − 0.08(5). The deviation of this trend from a 1 : 1 behaviour is not statistically significant and this, therefore, underlines the general assumption of the model that the nitrogen inclusion is virtually complete.

From a structural point-of-view, the simplification of the general formula from Zn_2−*x*_GeO_4−*x*−3*y*_N_2*y*_ to Zn_2−*x*_GeN_2_O_1−*x*_ also implies that one would not expect an extended amount of defects on either the cation or the anion sites.

With the formal splitting of the reaction product into ZnGeN_2_ and ZnO, as outlined above, the question poses as to whether this is simply a formalism, or may comport a true physical value. The latter could further be reasoned by the fact that ZnO crystallises in the wurtzite-type structure, and hence isostructurally to oxygen richer zinc germanium oxide nitride. Further, the lattice parameters of ZnO (*a* = 3.25 Å, *c* = 5.21 Å)^37^ at room-temperature are not too dissimilar to those of oxide rich zinc germanium nitrides (*e.g.* Zn_1.25_Ge_0.59_N_1.2_O_0.8_; *a* = 3.21 Å, *c* = 5.20 Å).^5^

### Structural features of the compounds

**Fig. 10:**
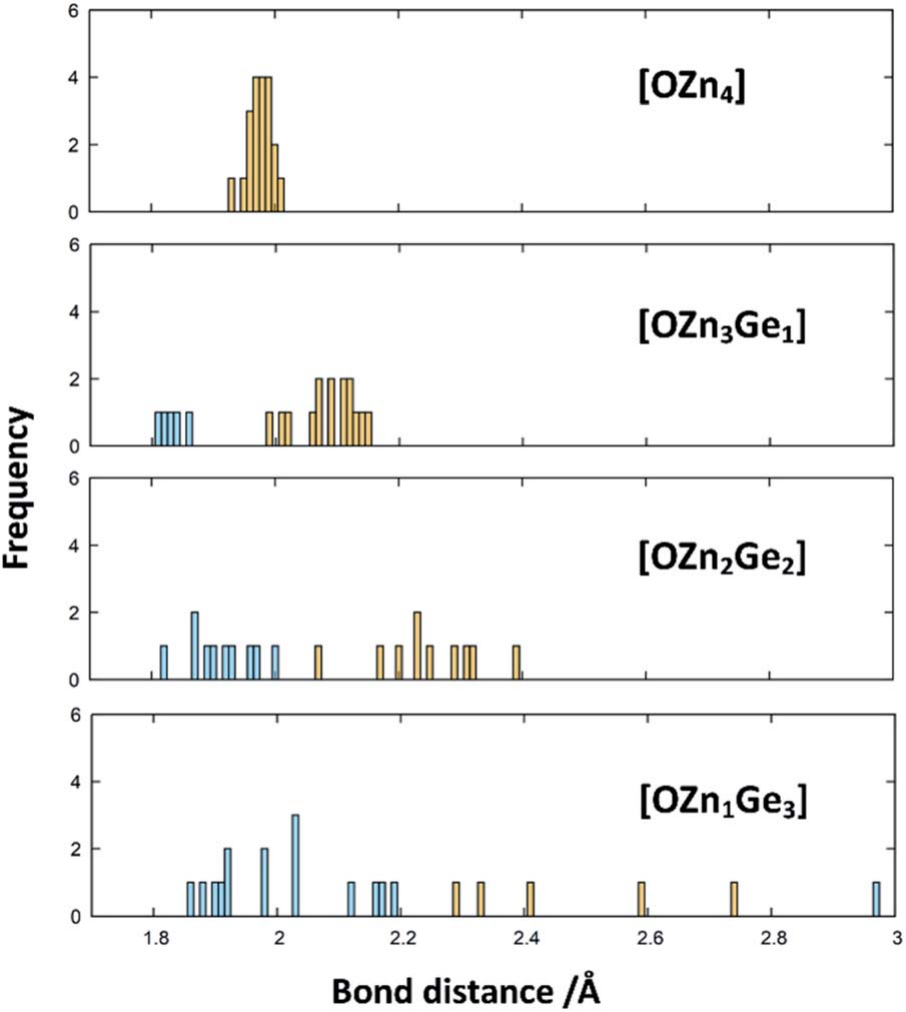
Histograms of the Zn–O (yellow) and Ge–O (blue) bond distances in the DFT-optimised structures. The histograms are ordered by the coordination environments of the oxygen atoms in the structures.

The X-ray diffraction patterns show no evidence of two distinct phases, however, even if they were isostructural in the wurtzite-type structure. Nonetheless, the formal separation in ZnGeN_2_ and ZnO is insofar useful as it may give a hint, why the Zn extrusion proceeds seemingly independent of the nitrogen inclusion. It is evident from the DFT calculations that oxygen energetically prefers the coordination with Zn, while the Ge atoms are preferably coordinated with nitrogen. Therefore, it is quite probable that zinc germanium oxide nitrides include local ordering in a way that there may be Zn and O richer regions and regions with a Zn : Ge ratio closer to 1 and a higher nitrogen content. The bonding distances are greatly influenced by the clustering of Zn and O (Fig. 10): When oxygen is solely surrounded by Zn, the optimised bond distances all exist in a narrow region in a range 1.9307 Å < *d*_Zn–O_ < 2.0077 Å. The more Ge enters the oxygen coordination, the more scattered become the bonding distances with the Ge–O distances generally lying below the Zn–O distances. Further, a Zn–O bond weakening expressed through longer distances can be observed when the bonding environment contains more Ge. Interestingly, the Ge–N and Zn–N distances are much less affected by the different environments (Fig. S2†), which is mainly due to the sheer predominance of these bonds against Zn–O and Zn–N bonds.

This is also seconded by the structural findings: the oxygen poor samples, *i.e.* those where Zn and O rich regions would be scarce, crystallise in the β-NaFeO_2_-type structure, while the oxygen richer samples crystallise in the wurtzite-type structure (Fig. 3). At first sight, one would expect anisotropic coordination tetrahedra around the Zn^2+^ and Ge^4+^ tetrahedra from a crystal chemical point-of-view, since both cations have different radii, and an ordered structure such as the β-NeFeO_2_-type structure would be preferred. The situation is insofar different for the oxygen richer samples, as the ordering is happening on a local level here, but diffraction probes a volume average of the crystal structure. The result of this averaging would be a disordered higher symmetry structure in the wurtzite-type structure as the aristotype of the β-NaFeO_2_-type structure.

### Prediction of reaction outcome

**Fig. 11:**
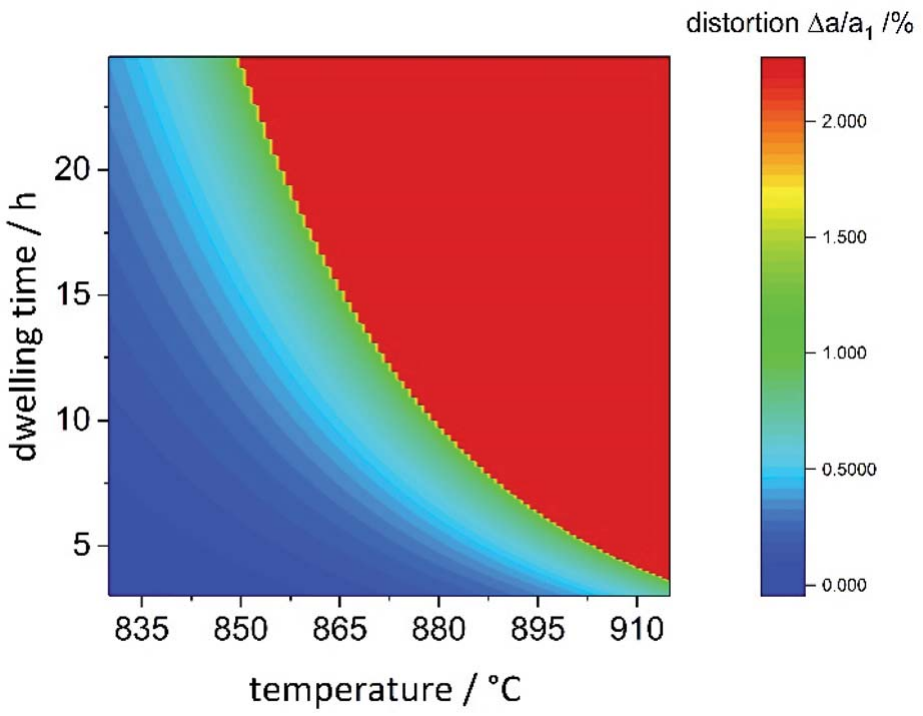
Heatmap of the orthorhombic distortion Δ*a*/*a*_1_*versus* dwelling time and reaction temperature. Full details of the reaction conditions may be found in the ESI.†

Combining the information on the chemical reaction with that on the structural transition, it is possible to model the reaction propagation in the dwelling time/reaction temperature parameter space based on the orthorhombic distortion as defined by the orthorhombic distortion parameter Δ*a*/*a*_1_ = (*a*_h2_ − *a*_h1_)/*a*_h1_ as defined previously. There is a clear relation between the lattice parameters *a*_o_ and *b*_o_ with the chemical composition (Fig. 5), and the orthorhombic distortion – as a value that is simple to obtain experimentally – may be taken as indication for the chemical composition. Since the orthorhombic distortion does not vary largely any more after having approached a plateau of 2.223%, this value was taken as a constant in well-ordered samples. Earlier stages of the reaction propagation were modelled using Arrhenius-type exponential functions both for reaction temperature and dwelling time (Fig. 11).

The temperature window, in which samples with maximal orthorhombic distortion and without significant amounts of side phases were obtained is relatively narrow, whereas the effect of the dwelling time appears to be less pronounced. While 20 hours reaction time at 865 °C were needed to reach maximal orthorhombic distortion, six hours at 910 °C were sufficient. One of the particularities we found in this synthesis is the seemingly abrupt change in the distortion in a narrow reaction condition range. For two samples with nominally identical reaction conditions (*T* = 880 °C, *t* = 10 h), we even experimentally obtained significantly different orthorhombic distortions of 1.00(7)% and 2.18(7)%. These reaction conditions mark the turning point and lie right at the edge of the red area of maximally distorted samples in Fig. 11. Minimal variations in the reaction conditions such as small fluctuations of the furnace temperature through differences in fume cupboard venting, which are not fully controllable may play a decisive role at the transition line. It would therefore be advisable to either prolong the reaction dwelling time or reaction temperature in order to avoid this degree of uncertainty.

## Conclusions

The reaction of Zn_2_GeO_4_ with NH_3_ is formally separated in two reaction steps, but we could clearly show that the two partial reactions proceed in a very specific scheme in that nitrogen is introduced in the structure first, before Zn and O are extruded. This finding offers a more systematic insight into the reaction and allows a more targeted synthesis of these materials. Given the anticipated importance of this class of materials as earth-abundant photovoltaic materials, this opens the possibility of systematic studies of cation disorder as a function of the oxygen content.

Our chemical study is seconded by structural investigations with X-ray powder diffraction that shows a structural change from the hexagonal wurtzite-type structure for oxygen richer zinc germanium oxide nitrides to the orthorhombic β-NaFeO_2_-type structure for the oxygen poorer zinc germanium nitrides. Our complementary DFT study suggests that this may be an effect of intimate intermixing of zinc and oxygen rich domains with zinc germanium nitride approximant domains on a strictly local level. We finally elucidated the optical bandgaps pf the materials and show their relationship to the composition. With bandgaps in the range of 2.7–3.4 eV, the zinc germanium oxide nitrides are probably not directly usable as photovoltaic material, but they form a potent model system to understand the general trends inherent to II–IV–N_2_ nitride materials. It will be most important to shine further light on the exact structural effects during this reaction, in order to fully understand the relationship between chemical composition, crystal structure and optoelectronic properties.

## Author contributions

Z. Y. W., J. B. and S. S. discussed the work and designed the work plan; Z. Y. W. performed the experimental preparation and preliminary data analysis; S. B. and M. L. performed the hot gas extraction analysis; D. F. performed the quantum chemical calculations with input from Z. Y. W.; J. B. wrote the manuscript with inputs from all authors.

## Conflicts of interest

There are no conflicts to declare.

## Supplementary Material

SC-012-D1SC00328C-s001
