# Supplementary material for: Elucidation of the reaction mechanism for the synthesis of ZnGeN2 through Zn2GeO4 ammonolysis
Source: Chem Sci. 2021 May 13;12(24):8493–500. doi: 10.1039/d1sc00328c (PMC8221196; doi:10.1039/d1sc00328c)
Supplement: SC-012-D1SC00328C-s001 [file SC-012-D1SC00328C-s001.pdf]

Electronic Supplementary Material (ESI) for Chemical Science.
This journal is © The Royal Society of Chemistry 2021

Supporting Information

Elucidation of the reaction mechanism for the synthesis of $ZnGeN_2$ through Zn_2GeO_4 ammonolysis

Zhenyu Wang,[a,b] Daniel Fritsch [a] Stefan Berendts,[c] Martin Lerch,[c] Joachim Breternitz*[a,d] and Susan Schorr*[a,b]

a Helmholtz-Zentrum Berlin für Materialien und Energie GmbH, Department Structure and Dynamics of Energy Materials, Hahn-Meitner-Platz 1, 14109 Berlin.

b Freie Universität Berlin, Department Geosciences, Malteserstraße 74-100, 12249 Berlin.

c Technische Universität Berlin, Fakultät II, Institut für Chemie, Straße des 17. Juni 135, 10623 Berlin, Germany

d Universität Potsdam, Mathematisch-Naturwissenschaftliche Fakultät, Karl-Liebknecht-Str. 24-25, 14476 Potsdam.

1) Synthesis conditions of the studied samples

Table S1: Summary of the reaction conditions for the samples studied herein

Sample number	Precursor mass /g	Reaction temp. /°C	dwelling time /h	gas flow for cooling /l·min^{-1}		Nominal Composition
1	6	835	10	1.5	N_2	$Zn_{1.91}GeO_{0.75}N_{2.10}$
2	6	880	10	0.05	NH_3	$Zn_{1.28}GeO_{0.18}N_{2.07}$
3	3	880	10	0.15		$Zn_{1.13}GeO_{0.09}N_{2.03}$
4	3	880	10	0.15		$Zn_{1.12}GeO_{0.03}N_{2.05}$
5	3	880	12	0.15		$Zn_{1.09}GeO_{0.03}N_{2.04}$
6	3	880	12	0.15		$Zn_{1.06}GeO_{1.03}N_{2.02}$
7	3	880	15	0.15		$Zn_{1.06}GeO_{1.02}N_{2.03}$

2) O and N contents as determined through hot gas extraction

Table S2: O and N contents as determined through hot-gas extraction and the respective sample masses.

Sample number	Oxygen / wt.-%	Nitrogen / wt.-%	Sample Weight / mg
1	5.97	15.6	9.01
	6.73	15.6	8.15
	6.50	15.7	9.12
4	0.410	23.3	8.31
	0.437	23.3	9.76
	0.492	23.1	7.78
7	0.285	23.5	9.29
	0.300	23.4	9.53
	0.337	23.5	8.82
6	0.395	23.3	8.79
	0.400	23.4	8.81

	0.384	23.1	9.42
3	1.05	21.3	9.67
	1.10	21.2	10.06
	1.00	21.2	10.07
2	2.12	21.3	8.80
	2.21	21.2	8.60
	2.12	21.2	8.64
5	0.394	23.3	8.96
	0.405	23.3	9.19
	0.459	23.3	8.69

3) DFT calculation details

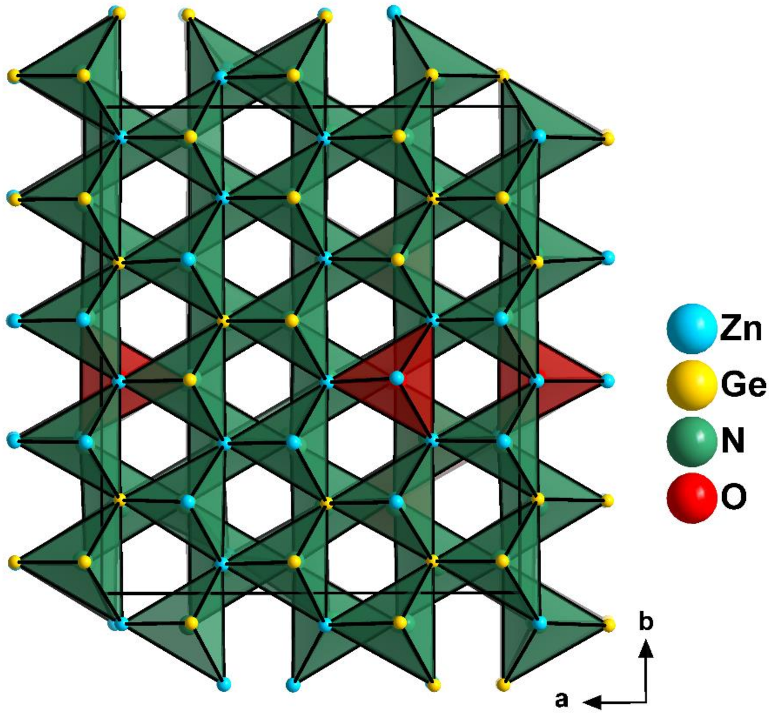

Figure S1: Representation of the DFT-optimised crystal structure of the supercell containing [OZn_4] tetrahedra along the crystallographic *c*-axis.

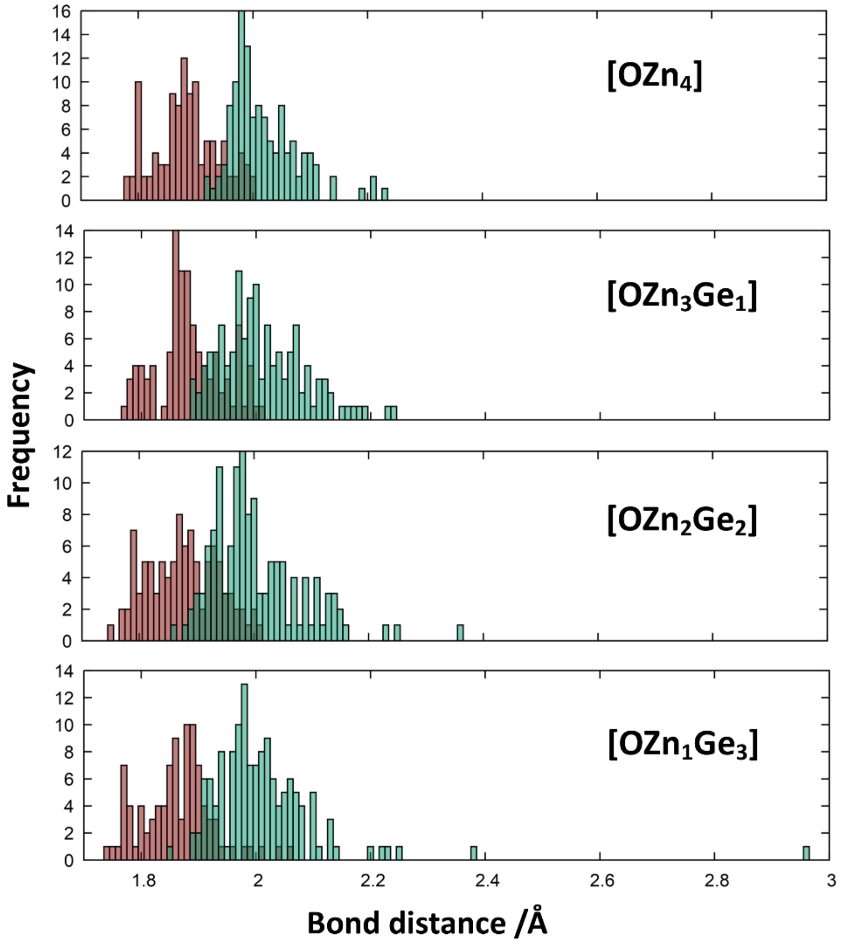

Figure S2: Histogram of the bond distance distributions in the DFT-optimised structures with Ge-N bonds in red and Zn-N distances in green.

4) Results of the Rietveld refinements

Sample Number 1:

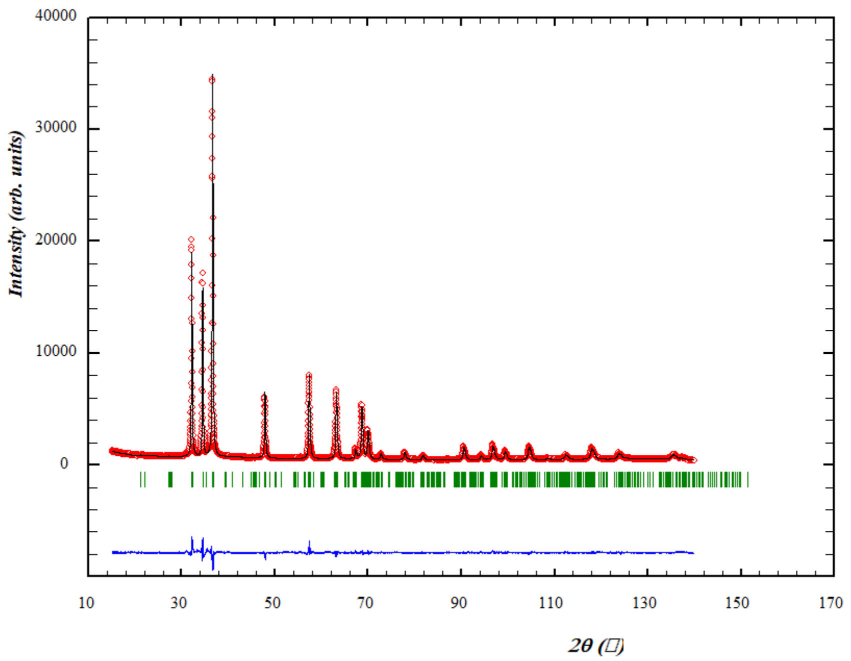

Figure S3: Plot for the Rietveld refinement of the X-ray powder diffraction profile: Measured Intensity (red circles), calculated intensity (black line), difference I_{obs}–I_{calc} (blue line) and calculated reflection positions (green ticks).

Table S3: Rietveld refinement summary

Orthorhombic, *Pna*2$_1$	c = 5.19767 (4) Å
Hall symbol: P 2c -2n	V = 185.63 (1) Å^3
a = 5.55805 (9) Å	X-ray radiation
b = 6.42574 (6) Å	
$2\theta_{min}$ = 15.09°, $2\theta_{max}$ = 139.97°, $2\theta_{step}$ = 0.02°	
R_p = 4.448	6322 data points
R_{wp} = 5.716	32 parameters
R_{exp} = 3.298	0 restraints
R_{Bragg} = 9.939	

Table S4: Fractional atomic coordinates and isotropic or equivalent isotropic displacement parameters for Sample 1

	x	*y*	*z*	B_{iso}
ZnGe	0.0911 (7)	0.1264 (15)	0.8729 (15)	0.68424
GeZn	0.0808 (8)	0.6249 (14)	0.86706	0.68424
N1	0.082 (6)	0.124 (7)	0.2668 (19)	0.68424
N2	0.07549	0.61405	0.23897	0.68424

Sample 2:

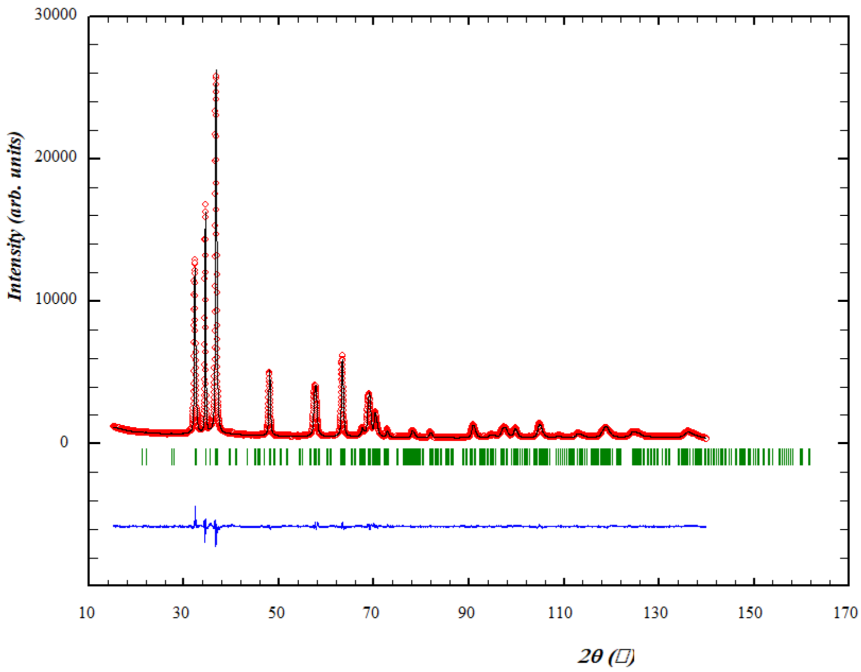

Figure S4: Plot for the Rietveld refinement of the X-ray powder diffraction profile: Measured Intensity (red circles), calculated intensity (black line), difference I_{obs}–I_{calc} (blue line) and calculated reflection positions (green ticks).

Table S5: Rietveld refinement summary

Orthorhombic, $Pna2_1$	c = 5.19389 (5) Å
Hall symbol: P 2c -2n	V = 183.79 (1) Å^3
a = 5.51540 (8) Å	X-ray radiation
b = 6.41566 (8) Å	
$2\theta_{min}$ = 15.12°, $2\theta_{max}$ = 140.00°, $2\theta_{step}$ = 0.02°	
R_p = 3.730	6245 data points
R_{wp} = 4.896	32 parameters
R_{exp} = 3.345	0 restraints
R_{Bragg} = 8.280	

Table S6: Fractional atomic coordinates and isotropic or equivalent isotropic displacement parameters for Sample 2

	x	y	z	B_{iso}
ZnGe	0.0879 (4)	0.124 (2)	0.8711 (16)	0.39809
GeZn	0.0756 (5)	0.626 (2)	0.86706	0.39809
N1	0.085 (4)	0.130 (5)	0.2531 (18)	0.39809
N2	0.07549	0.61405	0.23897	0.39809

Sample 3:

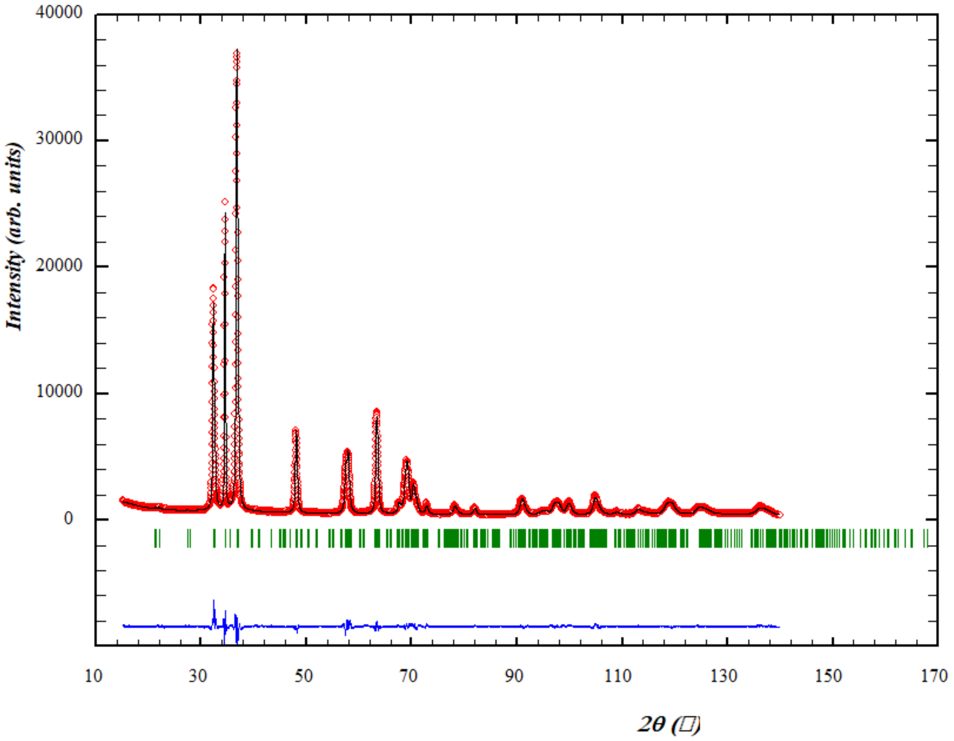

Figure S5: Plot for the Rietveld refinement of the X-ray powder diffraction profile: Measured Intensity (red circles), calculated intensity (black line), difference I_{obs}–I_{calc} (blue line) and calculated reflection positions (green ticks).

Table S7: Rietveld refinement summary

Orthorhombic, *Pna*2$_1$	c = 5.19289 (6) Å
Hall symbol: P 2c -2n	V = 183.30 (1) Å^3
a = 5.50173 (8) Å	X-ray radiation
b = 6.41593 (8) Å	
$2\theta_{min}$ = 15.07°, $2\theta_{max}$ = 139.95°, $2\theta_{step}$ = 0.02°	
R_p = 4.246	6322 data points
R_{wp} = 5.481	32 parameters
R_{exp} = 2.958	0 restraints
R_{Bragg} = 2.787	

Table S8: Fractional atomic coordinates and isotropic or equivalent isotropic displacement parameters for Sample 3

	x	y	z	B_{iso}
ZnGe	0.0865 (4)	0.1237 (15)	0.8729 (10)	0.33663
GeZn	0.0755 (4)	0.6260 (15)	0.86706	0.33663
N1	0.094 (3)	0.134 (4)	0.2553 (13)	0.33663
N2	0.07549	0.61405	0.23897	0.33663

Sample 4:

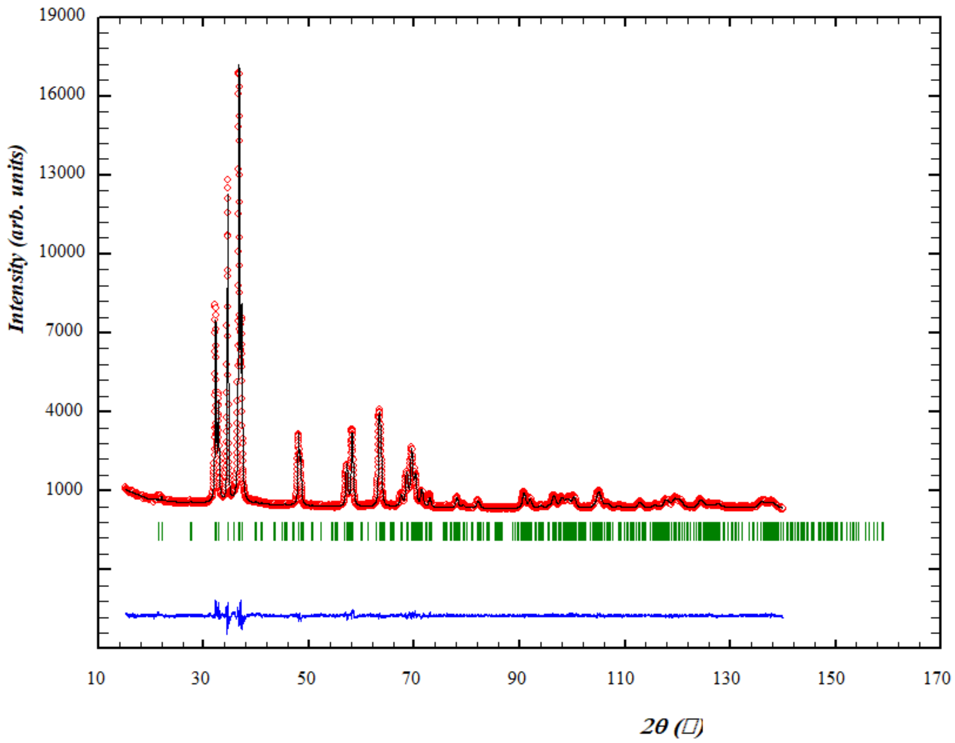

Figure S6: Plot for the Rietveld refinement of the X-ray powder diffraction profile: Measured Intensity (red circles), calculated intensity (black line), difference I_{obs}–I_{calc} (blue line) and calculated reflection positions (green ticks).

Table S9: Rietveld refinement summary

Orthorhombic, *Pna*2_1	c = 5.18922 (6) Å
Hall symbol: P 2c -2n	V = 182.72 (1) Å^3
a = 5.46291 (6) Å	X-ray radiation
b = 6.44537 (7) Å	
$2\theta_{min}$ = 15.11°, $2\theta_{max}$ = 139.99°, $2\theta_{step}$ = 0.02°	
R_p = 4.061	6322 data points
R_{wp} = 5.178	35 parameters
R_{exp} = 3.731	0 restraints
R_{Bragg} = 2.696	

Table S10: Fractional atomic coordinates and isotropic or equivalent isotropic displacement parameters for Sample 4

	x	y	z	B_{iso}
GeZn	0.0742 (4)	0.1279 (13)	0.8767 (10)	0.30676
ZnGe	0.0846 (4)	0.6238 (14)	0.86706	0.30676
N1	0.094 (3)	0.109 (6)	0.262 (2)	0.30676
N2	0.090 (3)	0.641 (6)	0.235 (3)	0.30676

Sample 5:

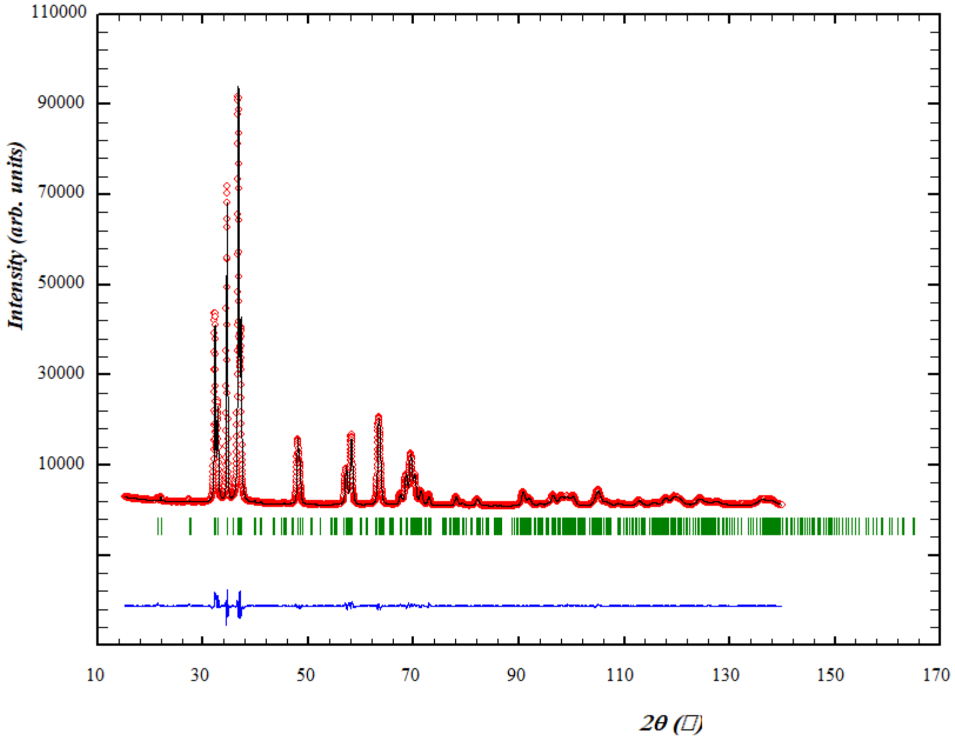

Figure S7: Plot for the Rietveld refinement of the X-ray powder diffraction profile: Measured Intensity (red circles), calculated intensity (black line), difference $I_{obs}–I_{calc}$ (blue line) and calculated reflection positions (green ticks).

Table S11: Rietveld refinement summary

Orthorhombic, *Pna*2_1	c = 5.18941 (4) Å
Hall symbol: P 2c -2n	V = 182.73 (1) Å^3
a = 5.46480 (5) Å	X-ray radiation
b = 6.44350 (5) Å	
$2\theta_{min}$ = 15.10°, $2\theta_{max}$ = 139.99°, $2\theta_{step}$ = 0.02°	
R_p = 3.495	6330 data points
R_{wp} = 4.547	35 parameters
R_{exp} = 1.872	0 restraints
R_{Bragg} = 1.926	

Table S12: Fractional atomic coordinates and isotropic or equivalent isotropic displacement parameters for Sample 5

	x	*y*	*z*	B_{iso}
GeZn	0.0748 (2)	0.1260 (4)	0.8712 (11)	0.33628
ZnGe	0.0849 (2)	0.6195 (4)	0.86706	0.33628
N1	0.0907 (16)	0.108 (2)	0.2649 (10)	0.33628
N2	0.0864 (17)	0.642 (2)	0.2243 (15)	0.33628

Sample 6:

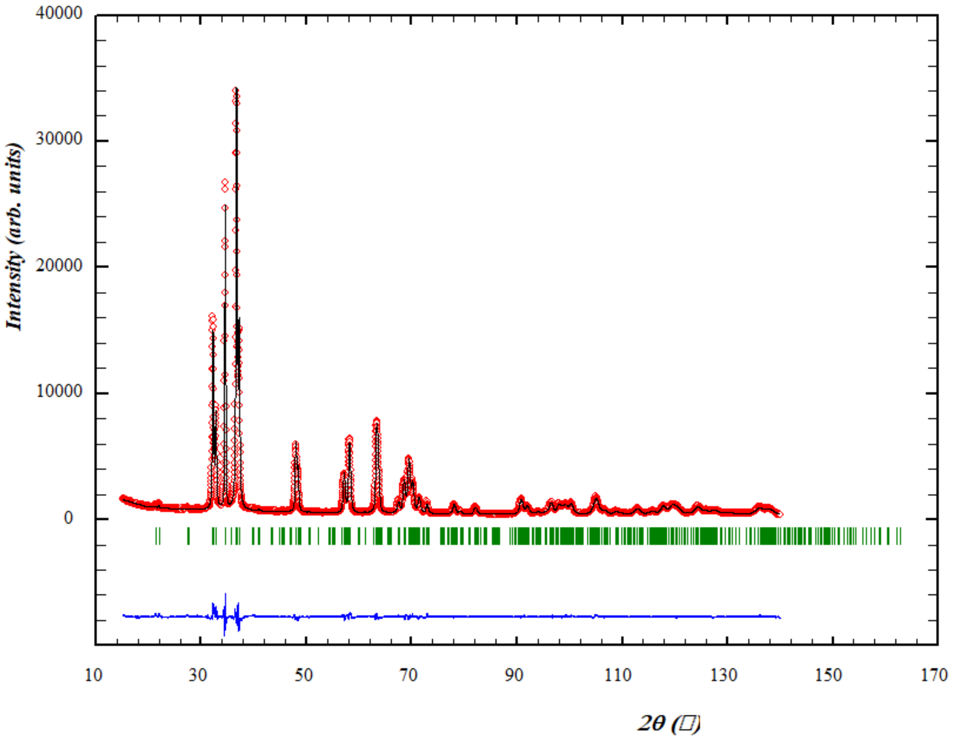

Figure S8: Plot for the Rietveld refinement of the X-ray powder diffraction profile: Measured Intensity (red circles), calculated intensity (black line), difference I_{obs}–I_{calc} (blue line) and calculated reflection positions (green ticks).

Table S13: Rietveld refinement summary

Orthorhombic, $Pna2_1$	c = 5.18926 (4) Å
Hall symbol: P 2c -2n	V = 182.73 (1) Å^3
a = 5.46315 (5) Å	X-ray radiation
b = 6.44544 (6) Å	
$2\theta_{min}$ = 15.08°, $2\theta_{max}$ = 139.97°, $2\theta_{step}$ = 0.02°	
R_p = 3.737	6322 data points
R_{wp} = 4.819	35 parameters
R_{exp} = 2.938	0 restraints
R_{Bragg} = 1.877	

Table S14: Fractional atomic coordinates and isotropic or equivalent isotropic displacement parameters for Sample 6

	x	y	z	B_{iso}
Ge1	0.0749 (3)	0.1292 (5)	0.8684 (14)	0.31165
Zn1	0.0845 (3)	0.6248 (6)	0.86706	0.31165
N1	0.0967 (18)	0.111 (3)	0.2647 (14)	0.31165
N2	0.086 (2)	0.645 (2)	0.2260 (16)	0.31165

Sample 7:

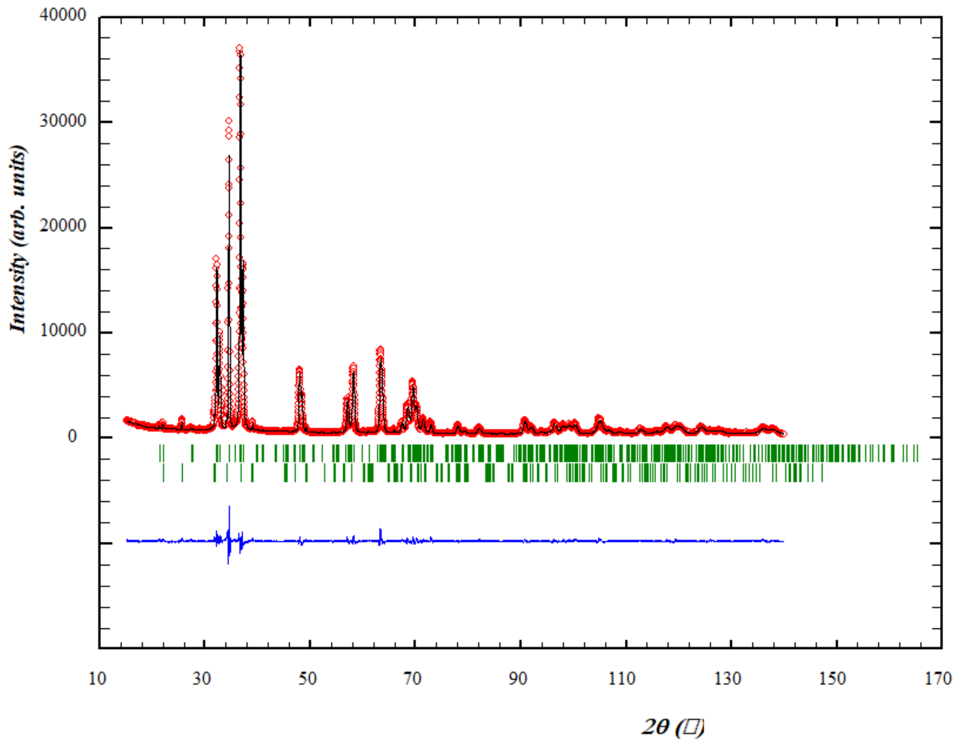

Figure S9: Plot for the Rietveld refinement of the X-ray powder diffraction profile: Measured Intensity (red circles), calculated intensity (black line), difference I_{obs}–I_{calc} (blue line) and calculated reflection positions (green ticks, Ge_3N_4 bottom, $Zn_{2-x}GeN_2O_{1-x}$ top).

Table S15: Rietveld refinement summary for the $Zn_{2-x}GeN_2O_{1-x}$ phase of Sample 7

Orthorhombic, $Pna2_1$	c = 5.18909 (5) Å
Hall symbol: P 2c -2n	V = 182.70 (1) Å^3
a = 5.45942 (6) Å	X-ray radiation
b = 6.44914 (7) Å	
$2\theta_{min}$ = 15.09°, $2\theta_{max}$ = 139.97°, $2\theta_{step}$ = 0.02°	Phase Fraction: 98.5(3) %
R_p = 4.639	6322 data points
R_{wp} = 6.095	35 parameters
R_{exp} = 2.936	0 restraints
R_{Bragg} = 2.529	

Table S16: Fractional atomic coordinates and isotropic or equivalent isotropic displacement parameters for the $Zn_{2-x}GeN_2O_{1-x}$ phase of Sample 7

	x	y	z	B_{iso}
GeZn	0.0855 (3)	0.1210 (7)	0.8605 (9)	0.17790
ZnGe	0.0721 (3)	0.6263 (9)	0.86706	0.17790
N1	0.098 (2)	0.104 (3)	0.256 (3)	0.17790
N2	0.082 (2)	0.640 (4)	0.233 (2)	0.17790

Table S17: Rietveld refinement summary for the Ge_3N_4 phase of Sample 7

DUMMY, $P6_3/m$	α = 90°
Hall symbol: -P 6c	β = 90°
a = 8.0309 (3) Å	γ = 120°
b = 8.0309 (3) Å	V = 171.92 (1) Å^3
c = 3.07807 (16) Å	X-ray radiation
$2\theta_{min}$ = 15.09°, $2\theta_{max}$ = 139.97°, $2\theta_{step}$ = 0.02°	Phase Fraction: 1.52(1) %
R_p = 4.639	0 data points
R_{wp} = 6.095	35 parameters
R_{exp} = 2.936	0 restraints
R_{Bragg} = 17.298	

Table S18: Fractional atomic coordinates and isotropic or equivalent isotropic displacement parameters for the Ge_3N_4 phase of Sample 7

	x	y	z	B_{iso}
Ge1	0.17120	-0.2342	0.25000	-0.01688
N1	0.33350	0.02950	0.25000	-0.01688
N2	0.33330	0.66670	0.25000	-0.01688

5) Dwelling time and reaction temperature relation samples

Only samples made under otherwise identical reaction conditions (250 K/h heating under 1.5 l/min N_2, dwelling and cooling under 0.15 l/min NH_3) were used to deploy the model. Ceramic foam was used to mantle holes where the reaction tube came in and out of the furnace in order to maximise the temperature stability during the reaction.

Scheme S1: The samples employed to develop the relationship between reaction temperature, dwelling time and orthorhombic distortion.

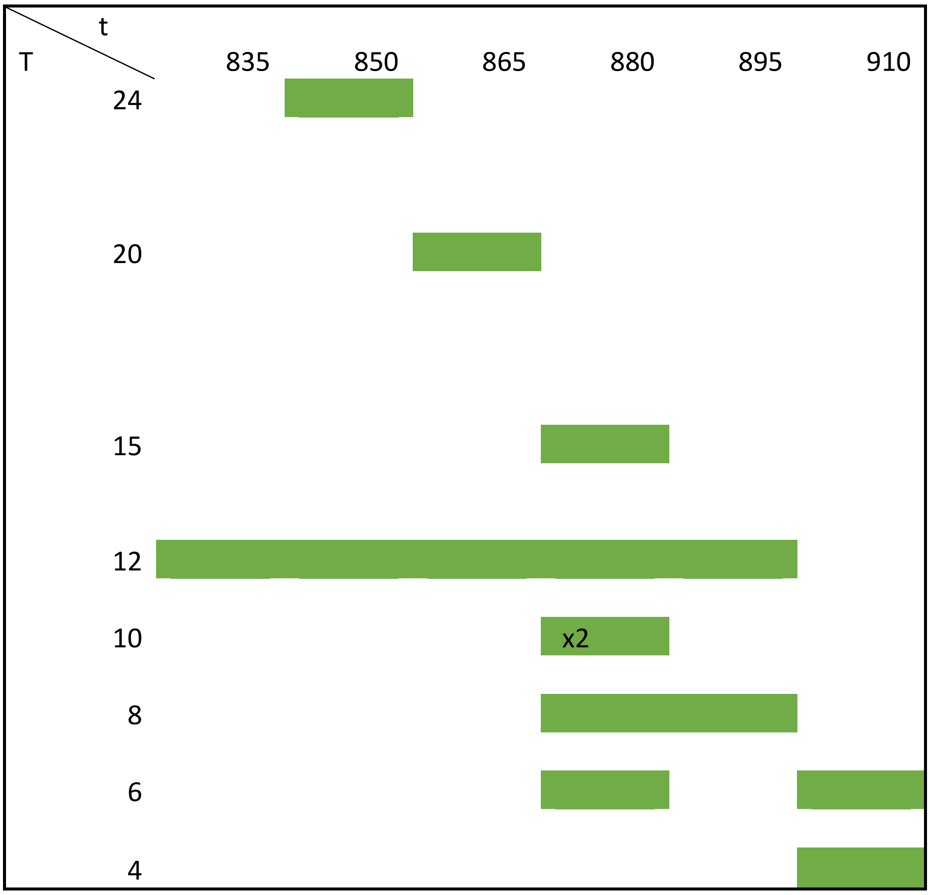

Mathematically, the model expresses as discontinuous function with:

for $\Delta a/a_1 \leq 1$:

$$\Delta a/a_1 = 0.353(0.235) \times \exp\{0.135(0.052) \times g(T) \times t\} - 0.353(0.235)$$

with

$$\ln[g(T)] = 33.929(1.5239) - 39106(1726)\frac{1}{T}$$

for $\Delta a/a_1 > 1$:

$$a/a_1 = 2.223(0.044)$$

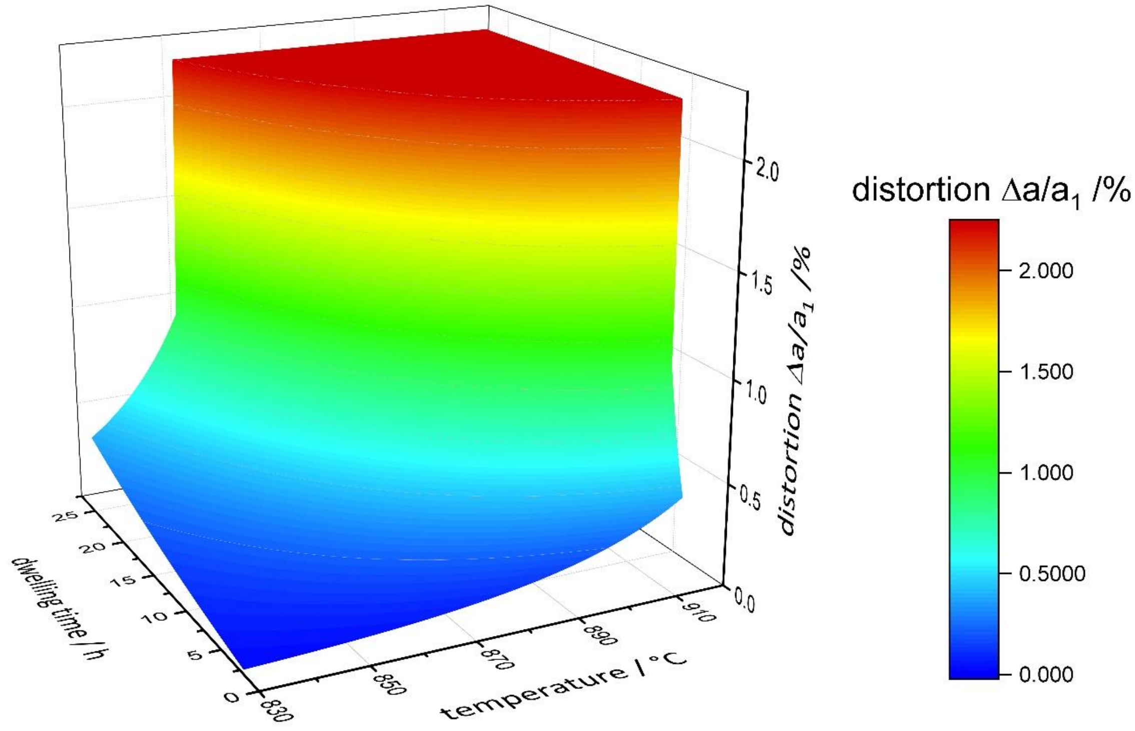

Figure S10: 3D surface plot of the orthorhombic distortion as a function of dwelling time and reaction temperature.
